# Homodimerization of RBPMS2 through a new RRM-interaction motif is necessary to control smooth muscle plasticity

**DOI:** 10.1093/nar/gku692

**Published:** 2014-07-26

**Authors:** Sébastien Sagnol, Yinshan Yang, Yannick Bessin, Fréderic Allemand, Ilona Hapkova, Cécile Notarnicola, Jean-François Guichou, Sandrine Faure, Gilles Labesse, Pascal de Santa Barbara

**Affiliations:** 1 INSERM U1046, Université Montpellier 1, Université Montpellier 2, 34295 Montpellier, France; 2 Centre de Biochimie Structurale, CNRS UMR5048, INSERM U1054, Universités Montpellier 1 et 2, 34295 Montpellier, France

## Abstract

In vertebrates, smooth muscle cells (SMCs) can reversibly switch between contractile and proliferative phenotypes. This involves various molecular mechanisms to reactivate developmental signaling pathways and induce cell dedifferentiation. The protein RBPMS2 regulates early development and plasticity of digestive SMCs by inhibiting the bone morphogenetic protein pathway through its interaction with *NOGGIN* mRNA. RBPMS2 contains only one RNA recognition motif (RRM) while this motif is often repeated in tandem or associated with other functional domains in RRM-containing proteins. Herein, we show using an extensive combination of structure/function analyses that RBPMS2 homodimerizes through a particular sequence motif (D-x-K-x-R-E-L-Y-L-L-F: residues 39–51) located in its RRM domain. We also show that this specific motif is conserved among its homologs and paralogs in vertebrates and in its insect and worm orthologs (CPO and MEC-8, respectively) suggesting a conserved molecular mechanism of action. Inhibition of the dimerization process through targeting a conserved leucine inside of this motif abolishes the capacity of RBPMS2 to interact with the translational elongation eEF2 protein, to upregulate *NOGGIN* mRNA *in vivo* and to drive SMC dedifferentiation. Our study demonstrates that RBPMS2 possesses an RRM domain harboring both RNA-binding and protein-binding properties and that the newly identified RRM-homodimerization motif is crucial for the function of RBPMS2 at the cell and tissue levels.

## INTRODUCTION

Fully mature vascular, visceral or digestive smooth muscle cells (SMCs) exhibit extensive plasticity in response to exogenous/endogenous stimulation or injury. This phenomenon implies dedifferentiation of contractile and functional SMCs into proliferative and migratory cells (1). Strong evidences suggest that SMC plasticity plays a crucial role during mammalian embryo development and in a large number of human diseases (2). Modulation of SMC dedifferentiation and remodeling of smooth muscle tissues is achieved through tight and long-term control of several signaling pathways, usually at the post-transcriptional level (2,3,4). This regulation is generally performed at multiples steps of ribonucleic acid (RNA) metabolism, such as during RNA transport and sub-cellular localization, splicing, translational regulation and degradation (5), and can involve specific regulatory RNAs, such as micro-RNAs (miR), and regulatory proteins, such as RNA-binding proteins. For instance, convergent studies demonstrated that microRNA 145 (miR-145) regulates the differentiation of gastrointestinal smooth muscle (6). Specifically, miR-145 controls directly and positively the expression of MYOCARDIN, which, in turn, regulates many steps of SMC differentiation (7,8). In parallel, RNA-binding protein for multiple splicing-2 (RBPMS2) also plays a role in the development and plasticity of digestive SMCs (9). RBPMS2 is an early marker of gastrointestinal smooth muscle precursor cells (9,10), which positively regulates mRNA expression of *NOGGIN*, the major inhibitor of the bone morphogenetic protein (BMP) pathway, through the formation of a *NOGGIN–*RBPMS2 ribonucleoprotein complex (9). Ectopic RBPMS2 expression in differentiated digestive SMCs hinders their ability to contract and induces their proliferation leading to their dedifferentiation, demonstrating that RBPMS2 expression has to be tightly regulated to avoid SMC dedifferentiation, as observed in chronic intestinal pseudo-obstruction syndrome (CIPO) and gastro-intestinal stromal tumors (GISTs) (9,11).

In agreement with the ability of RBPMS2 to bind *NOGGIN* mRNA and regulate its expression, RBPMS2 belongs to the superfamily of RNA recognition motif (RRM)-containing proteins. Its RRM domain appears to be conserved in vertebrates and insects, where its *Drosophila melanogaster* homolog couch potato (CPO) regulates reproductive diapause (12). Sequence conservation of the common RRM motif extends to the N-terminal RRM of mechanosensory-defective mutant gene-8 (MEC-8), a developmental protein involved in the regulation of alternative splicing in *Caenhorabditis elegans* (13). Based on sequence similarities and the fact that all three proteins are involved in organ development (muscles and neuron), a conserved mechanism of action was suggested. Surprisingly, RBPMS2 and some CPO have only one RRM domain, while MEC-8 and other CPO contain two RRMs in tandem. This difference suggests that additional regulations and/or different supramolecular organizations are necessary for the specific interaction of RBPMS2 with its target mRNA. Indeed, it is now well established that the RRM core most often recognizes only two or three nucleotides (14,15). This corresponds to a rather narrow interface to specifically recognize a target RNA among all those present in a cell. Spatiotemporal regulation of RBPMS2 expression may also help in targeting the right RNAs, but additional features might be necessary to ensure specificity. Alternatively, homo- or hetero-polymerization may also promote the formation of a larger interface to facilitate the specific recognition of RNA by the resulting complex (14).

The aim of this study was to determine the structural features and the molecular mechanisms through which RBPMS2 controls the plasticity of digestive smooth muscle. By combining distinct complementary and multidisciplinary approaches, we show that RBPMS2 homodimerizes *in vitro* in cell culture and in animals. Therefore, we identified a particular sequence motif promoting homodimerization of RRM domain that is conserved also in CPO and MEC-8. We also demonstrate that this newly identified RRM-homodimerization motif (residues 39–51) is crucial for the function of RBPMS2 during SMC dedifferentiation.

## MATERIALS AND METHODS

### Constructs and cell line

Human *RBPMS2* cDNA was used as template (11). The substitution of L49 by E or Q in human RBPMS2 was done with the QuikChange site-directed mutagenesis kit and protocol (Stratagene). The complementary deoxyribonucleic acid (cDNA) sequence coding amino acids 27–117 of human *RBPMS2* and mutated L49E were subcloned into pET22 (pET22-*RBPMS2*-Nter and pET22-*RBPMS2*-Nter-L49E). Full-length human *RBPMS2*, *RBPMS2*-L49E and *RBPMS2*-L49Q were subcloned in the pCS2 vector with an in-frame N-terminal HA tag and the cytomegalovirus (CMV) promoter (pCS2-HA-*RBPMS2*; pCS2-HA-*RBPMS2*-L49E and pCS2-HA-*RBPMS2*-L49Q, respectively). Full-length human *RBPMS2* and *RBPMS2*–L49E were subcloned in the pHRTK vector with an in-frame N-terminal Myc tag and the CMV promoter (pHRTK-Myc-*RBPMS2* and pHRTK-Myc-*RBPMS2*-L49E, respectively). The cDNA sequence coding amino acids 621–838 of human *eEF2* (identified in a yeast two-hybrid (Y2H) screen as interacting with RBPMS2) was subcloned in the pCS2 vector with an in-frame N-terminal HA tag and the CMV promoter (pCS2-HA-*eEF2*). HA-tagged human *TC10* was previously described (16). Myc-tagged chick full-length *RBPMS2* with the L40E mutation (obtained with the QuikChange site-directed mutagenesis kit) was cloned into the RCAS vector to produce the RCAS-Myc-*RBPMS2-*L40E plasmid. Myc-tagged chick full-length *RBPMS2* (RCAS-Myc-*RBPMS2*), *GFP* (RCAS-*GFP*) and Myc-NICD were previously described (9,10,17). All plasmids were checked by DNA sequencing, protein expression and expression plasmids were transfected in the avian DF-1 chicken fibroblast cell line (ATCC-LGC) or human embryonic kidney 293 (HEK293) cell line as previously described (11,18).

### Protein expression and purification

pET22-*RBPMS2*-Nter and pET22-*RBPMS2*-Nter-L49E were transformed in *Escherichia coli* BL231DE3 cells and grown on LB agar with 100-μg/ml ampicillin at 37°C overnight. The next day, colonies were resuspended in expression media (ZYM 5052 or N5052 for ^15^N-labeled proteins) (19). For ^13^C/^15^N isotopic labeling, proteins were expressed in M9 medium in a fermenter with ^13^C-glucose and 15N-NH4Cl as only sources of carbon and nitrogen. Cells were harvested by 15-min centrifugation at 6000g at 4°C, resuspended in 50-mM Tris pH8, 300-mM NaCl and 1-mM dithiothreitol (DTT) (buffer A) and stored at −40°C. Cells were lysed by sonication and insoluble proteins and cell debris were sedimented by centrifugation at 40000g at 4°C for 30 min. Supernatants were filtered through 0.45-μm filters and loaded onto affinity columns (His Trap FF, GE Lifescience), equilibrated with buffer A. Columns were washed with 20 column volume of buffer A and proteins eluted with a linear 0–100% gradient of buffer B (buffer A containing 0.5-M imidazole). The peak fractions were analyzed by sodium dodecyl sulphate-polyacrylamide gel electrophoresis (SDS-PAGE). Fractions containing RBPMS2-Nter or RBPMS2-Nter-L49E were pooled and concentrated if needed.

### Nuclear magnetic resonance spectroscopy

All nuclear magnetic resonance (NMR) experiments were carried out at 305K on a Bruker Avance III 700 spectrometer equipped with a 5-mm z-gradient TCI cryoprobe, using the standard pulse sequences (20). NMR samples consisted of ∼0.8 mM 15N- or 15N/13C-labeled proteins dissolved in 25-mM phosphate buffer, 50-mM NaCl, pH 6.3 with 5% D2O as an internal lock. ^1^H chemical shifts were directly referenced to the methyl resonance of 4,4-dimethyl-4-silapentane-1-sulfonic acid (DSS), while ^13^C and ^15^N chemical shifts were referenced indirectly to the absolute 15N/1H or 13C/1H frequency ratios. All NMR spectra were processed and analyzed with GIFA (21). To distinguish inter- from intra-molecular nuclear Overhauser effects (NOEs), we prepared a heterolabeled dimer by mixing equivalent amounts of non-labeled and fully (^13^C/^15^N-) labeled proteins and then analyzed the mixture unfolding/refolding process. In brief, 4 mg of non-labeled and labeled proteins were added to 4 ml of 8.0-M urea solution and dialyzed against 2 l of 50-mM phosphate buffer (pH 6.3) containing 50-mM NaCl. The sample was then concentrated to 0.5 ml and washed twice with the dialysis buffer to eliminate all urea traces. The concentrated sample was lyophilized and dissolved in 100% D_2_O. Proper refolding was checked using ^1^H-1D and ^1^H, ^13^C 2D NMR spectroscopy. The ^13^C F1-filtered, F2-edited NOESY spectra were recorded. The filtered spectrum of the aromatic/aliphatic region was a special case, thus a frequency list of the ^13^C nuclei was added to the original pulse program. The ^13^C transmitter was set to 40 ppm at the center of the aliphatic ^13^C resonance before the NOESY mixing time and shifted to 125 ppm in the aromatic field during the edition period. Accordingly, the coupling constants were adjusted to 145 Hz and 160 Hz, respectively, during the two periods.

### Structure calculations

The ^1^H-^1^H distance restraints were derived from the NOESY spectra, all with 100-ms mixing time. NOE intensities were converted into inter-proton distances: 2.4 Å (for very strong intensities), 2.8 Å (strong), 3.6 Å (medium), 4.4 Å (weak) and 5.0 Å (very weak). Pseudo-atoms to replace methyl and degenerated methylene protons were introduced and inter-proton distances were corrected using standard criteria. Based on H/D exchange experiments, hydrogen-bond restraints were used for the residues in the regular secondary structures. When identified, the hydrogen bond was enforced using two distance restraints: *d*(NH,O) = 1.8–2.3 Å and *d*(N,O) = 2.7–3.3 Å. The backbone dihedral angle restraints Φ and Ψ were derived from chemical shift analysis using the PREDITOR program (22). Side chain dihedral angle restraints (χ^1^, χ^2^) were derived from detailed analysis of the proton NOE network and inspection of COSY/TOCSY peak intensities between Hα/Hβ protons, taking one of these three values: −60°, 60° or 180°. In this study, 19 such restraints (per monomer) from 18 residues showing no ambiguities were used in the final calculation. For the structure calculation of RBPMS2-Nter, beside 1210 (per monomer) classical intra-molecular distance restraints taken from 2D/3D-NOESY spectrum, 43 other ones derived from filtered NOESY spectrum were used as inter-molecular restraints. Two hundred structures were calculated using the CYANA-2.1 program (23) for RBPMS2-Nter. The thirty structures with the lowest target function values were further energy-minimized with CNS 1.2 according to the RECOORD procedure (24) and analyzed with INSIGHT and PROCHECK (25). The rms deviations were calculated with MOLMOL (26) and all statistics are in Table 1.

**Table 1.** 

NMR distance and dihedral constraints	
Distance restraints:	
Intra-molecular	2420
Intra-residue	486
Inter-residue	
Sequential (|*i* − *j*| = 1)	706
Medium-range (1>*i* − *j*| < =4)	416
Long-range (|*i* − *j*| >=5)	772
Hydrogen bonds	40
Inter-molecular	86
Dihedral angle restraints:	
ϕ	132
ψ	138
χ_1_, χ_2_	38
Total experimental restraints:	2814
Structure statistics	
Violations:	
Distance constraint violation (>0.3Å)	0
Dihedral angle violation (>5°)	0
Deviations from idealized geometry:	
Bond lengths (Å)	0.0118 ± 0.0004
Bond angles (°)	1.2996 ± 0.0354
Impropers (°)	1.5793 ± 0.0548
Ramachandran plot^a^(%)	
Most favored region	90.5
Additionally allowed region	9.5
Generously allowed region	0.0
Disallowed region	0.0
Average pairwise rms deviation^a^ (Å)	Residues (29A-112A, 29B-112B)
Backbone	0.84 ± 0.11
Heavy	1.52 ± 0.12

^a^Pairwise rms deviation was calculated among 15 refined structures. The Ramachandran plot was calculated to the lowest-energy structure.

### Small-angle X-ray scattering experiments

The small-angle X-ray scattering (SAXS) measurements for RBPMS2-Nter and the RBPMS2-Nter-L49E proteins were carried out at the EMBL BioSAXS beamline P12 at the Petra III Storage Ring, DESY (Hamburg, Germany) using an X-ray wavelength of 1.24 Å and a sample-to-detector distance of 3.1 m. Samples were measured at 10°C at two concentrations (4.2 and 1.9 mg/ml for RBPMS2-Nter and 2.2 and 1.1 mg/ml for RBPMS2–Nter-L49E) in a buffer containing 50-mM Tris pH8, 300-mM NaCl and 1-mM DTT. The scattering patterns of the corresponding buffer solutions were recorded before and after the measurements of the protein sample. The scattering profiles measured covered a momentum transfer range of 0.002 < *s* < 0.45 Å^−1^ (with *s* = 4πsin(θ)/λ). Inspection of the consecutive 20-s X-ray exposures discarded the presence of radiation damage. Final curves at each concentration were derived after the averaged buffer patterns were subtracted from the protein ones using standard protocols with PRIMUS (27). No signature of aggregation was observed in any of the curves. The forward scattering, *I(0)*, and the radius of gyration, *R_g_*, were evaluated using the Guinier's plot, assuming that at very small angles (*s.Rg* < 1.3). The agreement between the SAXS curve and the theoretical NMR structure was evaluated with CRYSOL (28) using a momentum transfer range of 0.010< *s* <0.45 Å^−1^.

### Size-exclusion chromatography with multi-angle laser light scattering

For determination of the state of these proteins, human RBPMS2-Nter and RBPMS2-Nter-L49E were analyzed using an HPLC-MALS apparatus. We loaded 0.05-ml samples of protein at ∼2–4 mg/ml onto a Superdex 75 10/300 gel-filtration column equilibrated at 0.5 ml/min with a mobile phase consisting of 20-mM Tris–HCl at pH 8 and 300-mM sodium chloride. The eluate was passed successively through a UV 900 GE monitor, a Wyatt Optilab rEX refractive index monitor and a Wyatt miniDawn TREOS 3-angle light-scattering detector with the system driven by an AKTA purifier HPLC system comprising a P900 pump. The data were processed, and molecular masses were calculated using the Astra 6.1 software (Wyatt).

### Y2H screening

Y2H screening was performed by Hybrigenics Inc. using the mating methodology as previously described (29). Briefly, a bait corresponding to amino acids 1–209 of human RBPMS2 was generated by fusion to LexA in the vector pB27 to screen a human placenta cDNA library, cloned in the pP6 plasmid, in the presence of 1 mM of 3-aminotriazole (3AT). The confidence of the tested interactions (*n* = 67 million) was scored using the Predicted Biological Score program to exclude false-positive results and promiscuous interactions.

### In situ proximity ligation assay

For the DuoLink *in situ* proximity ligation assay (PLA) (30), DF-1 cells or primary SMCs transfected with different plasmid combinations were labeled with mouse anti-HA (Santa Cruz Biotechnologies) and rabbit anti-Myc (Ozyme) antibodies and incubated with a pair of nucleotide-conjugated secondary antibodies (rabbit PLA probe MINUS and mouse PLA probe PLUS; OLINK Biosciences, Uppsala Sweden) in saturation solution (phosphate buffered saline (PBS), 0.1% Tween, 5% Normal Donkey Serum). In addition, secondary mouse and rabbit anti-IgG coupled to Alexa 488 and 555 (Invitrogen) were also used to detect protein expression. Both Minus and Plus PLA probes interact with a rolling-circle nucleotide template when the distance between them is less than 40 nm. These complexes were ligated in the presence of a ligase in hybridization solution. The circular template was then amplified using a polymerase, while far-red-labeled probes hybridized the amplified sequence, according to the manufacturer's instructions. *In situ* PLA images were acquired using a Carl-Zeiss LSM710 confocal microscope.

### Immunoprecipitation and glutaraldehyde crosslinking

DF-1 cells were lysed in lysis buffer (20-mM Tris pH8, 50-mM NaCl, 1% NP40, cOmplete ethylenediaminetetraacetic acid (EDTA)-free Protease Inhibitor Cocktail (Roche)). For immunoprecipitation assays, 50 μg of total protein lysates were incubated in immunoprecipitation buffer (50-mM Tris pH8, 150-mM NaCl, 0.4% NP40, cOmplete, EDTA-free Protease Inhibitor Cocktail (Roche)) with rabbit anti-Myc antibodies (Ozyme) pre-adsorbed to protein A-Sepharose CL-4B (GE Healthcare) at 4°C for 1 h and washed extensively. For glutaraldehyde crosslinking, 4 μg of GFP-, Myc-RBPMS2 and Myc-RBPMS2-L410E-expressing DF1 total protein extracts were incubated at 4°C in 36 μl of 0,1% PBS-buffered glutaraldehyde solution (Sigma-Aldrich) during 10 or 30 s and reaction was stopped with 4 μl of Tris 1M pH8. Protein samples were boiled in SDS-PAGE sample buffer, separated by 12% SDS-PAGE and transferred to nitrocellulose membranes. Membranes were blocked with 10% nonfat milk in TBS/0.1% Tween and probed with mouse anti-HA (InvivoGen), rabbit anti-Myc (Sigma), rabbit anti-GFP (Torrey Pines Biolabs) or rabbit anti-eEF2 (Abcam) polyclonal antibodies overnight. After several washes, membranes were incubated with the relevant horseradish peroxidase-conjugated secondary antibodies (Perkin Elmer). Detection was performed by chemiluminescence (Santa Cruz Biotechnologies) on Kodak films.

### Fluorescence anisotropy and gel mobility shift assays (electrophoretic mobility shift assay)

Human RBPMS2-Nter and RBPMS2-Nter-L49E were labeled with the N-Hydroxysuccinimide (NHS) ester of ATTO647N in 20-mM Na-phosphate buffer pH 7.5 with 50-mM KCl at room temperature for 3 h. Labeled proteins were separated from the free dye using 2-ml Zeba spin desalting columns (Thermo Scientific) equilibrated in binding buffer (20-mM Tris-HCl pH 7.5, 100-mM KCl). Anisotropy measurements were carried out at 25°C in dilution mode. RBPMS2-Nter–ATTO647N and RBPMS2-Nter-L49E-ATTO647N (2-nM final) were then mixed with different RNAs (2-μM final) in binding buffer. Mixtures were then serially diluted in binding buffer containing 2-nM RBPMS2-Nter-ATTO647N or RBPMS2-Nter-L49E-ATTO647N in Corning black 384-wells assay plates. Measurements were made with a TECAN Safire2 apparatus in polarization mode. DNA templates were constructed by polymerase chain reaction using the pCMV6-XL5 plasmid that contains the human *NOGGIN* cDNA including the 5′ and 3′ untranslated regions and the open reading frame (OriGene) and forward primers with the T7 promoter sequence. These templates were used to synthesize *in vitro* human *NOGGIN* RNA with the T7 high yield RNA synthesis Kit (New England Biolabs). For electrophoretic mobility shift assay (EMSA) experiments, 100 nM of 161-nt *NOGGIN* RNA (570–730 nucleotides from *NOGGIN* mRNA) in Tris 20 mM pH7.4, KCl 100 mM EDTA 1 mM, bovine serum albumin (BSA) 10 μg/ml, DTT 1 mM, RNaseOUT 1 U/μl (Invitrogen), Glycerol 2% was mixed with the recombinant RBPMS2-Nter or RBPMS2-Nter-L49E (from 0.1 to 5 μM). All reaction components were incubated at 20 ± 1°C for 30 min and were loaded on 10% polyacrylamide gels (19:1 acrylamide:bis) in Tris-Borate-EDTA 1X buffer and run at 10 V/cm at 4°C for 3–4 h. The gel was visualized with SYBR^®^ Green EMSA nucleic acid gel stain (Molecular Probes) for RNA and SYPRO^®^ Ruby protein gel stain (Sigma-Aldrich) for proteins. Images were obtained on Chemidoc transilluminator (BioRad) and quantification of bound RNA was made with FIJI (ImageJ) software. Fraction of bound RNA was calculated in a first step by dividing the intensities of mobility-shifted bands (corresponding to the protein–RNA complexes) by the sum of intensities of both free and bound RNA, and in a second step by weighting the result by the ratio between of the sample intensity and the protein-free sample intensity. The fraction of bound RNA was plotted against the protein concentration and fitted to the equation

\begin{equation*} {\it y} = {\rm m}1 + ({\rm m}2 - {\rm m}1)/[1 + ({\it x}/{\rm m}3)^{{\rm m}4} ], \end{equation*}

where *y* = fraction of bound RNA, *x* = protein concentration, m1 = maximum fraction bound RNA, m2 = minimum fraction bound RNA, m3 = *K*_d_ and m4 = cooperativity, using the software KaleidaGraph version 4.5 (Synergy Software) as previously reported on RRM-containing proteins (31).

### Avian retroviral misexpression system and analysis

Fertilized White Leghorn eggs from Haas Farm (France) were incubated at 38°C in humidified incubators. Gastrointestinal tissues from chick embryos were dissected as described (18). Retroviral constructs were transfected in DF-1 cells to produce retroviruses. Retroviruses were injected into the splanchnopleural mesoderm of Stage-10 chicken embryos to target the stomach mesenchyme (9,18). Eggs were then placed at 38°C until harvested. *In situ* hybridization experiments on stomach were carried out as described (10). Antisense *NOGGIN* riboprobes were generated using the chick *NOGGIN* template (9) by reverse transcription with incorporation of digoxigenin-UTP (Roche). Anti-digoxigenin antibodies coupled to alkaline phosphatase (Roche) were used to detect *NOGGIN* mRNA/antisense probe complexes with the BM Purple solution (Roche). Images were acquired using a Nikon-AZ100 stereomicroscope.

### Primary SMC cultures

Primary cell cultures from E15 gizzard muscle were prepared as described (9). Briefly, the tunica muscularis was carefully separated from the serosa and tunica mucosa before collagenase dissociation. Isolated cells were cultured in Dulbecco's modified Eagle's medium with 0.2% BSA and 5-μg/ml insulin on Matrigel-coated plates to maintain cell differentiation (more than 95% of isolated cells were Desmin- and αSMA-positive). Differentiated SMCs were then infected or electroporated with Neon Transfection System (Invitrogen) with different expression constructs and analyses. For immunodetection, anti-Myc (Ozyme), anti-HA (Santa Cruz Biotechnologies), anti-Calponin (Sigma-Aldrich) and anti-Phospho-Histone H3-Ser10 (Millipore) antibodies were used. Nuclei were stained with Hoechst (Molecular Probes). Images were acquired using a Carl-Zeiss AxioImager microscope.

## RESULTS

### RBPMS2 sequence analysis and molecular modeling

**Figure 1.**
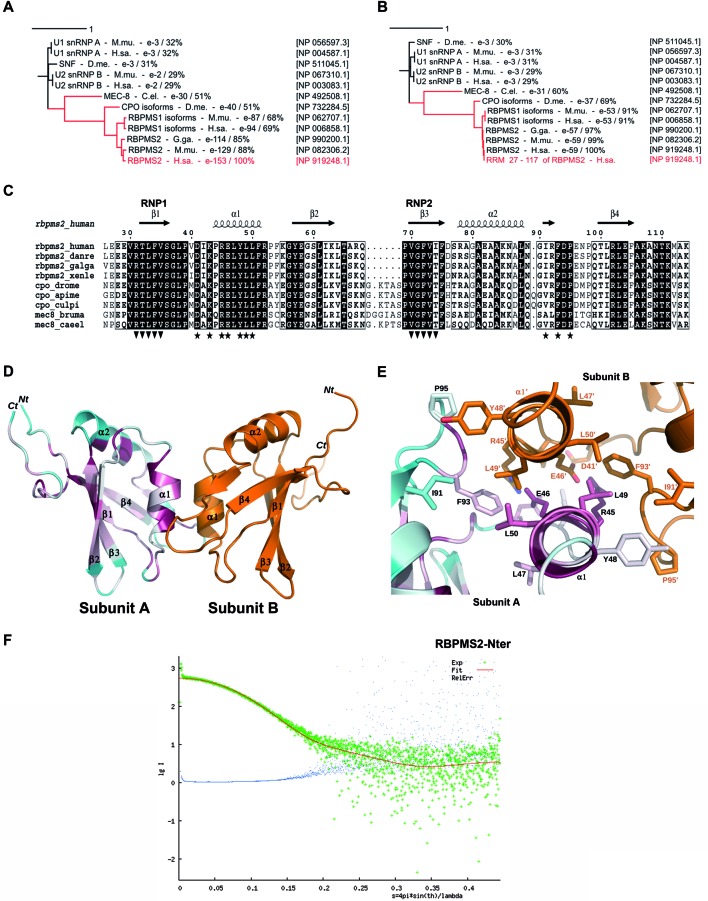
Dimeric structure of RBPMS2 in solution. (**A, B**) RBPMS2, CPO and MEC-8 proteins harbor evolutionary conserved RNA recognition motif (RRM) domain. (A) Phylogenetic tree using human RBPMS2 protein as query (in red). (B) Phylogenetic tree using the RBPMS2 RRM domain (amino acids from 27 to 117) as query (in red). After each leaf end follow the protein names, the species names (H.sa.: Human; M.mu.: *Mus musculus*; G.ga.: *Gallus gallus*; D.me.: *Drosophila melanogaster*; C.el.: *Caenhorabditis elegans*), the E-value and the percentage of identity regarding the query. The accession number of each protein is presented on the right column of the figure. Scale bar represents evolution distance of leaf branches (A.U.). The figure was prepared using iTOL (http://itol.embl.de). (**C**) Sequence-structure alignment of the RRM domain in RBPMS2 homologs. The N-terminus (residues 27–117) of human RBPMS2 was aligned with the corresponding segments in the sequences of RBPMS2, CPO or MEC-8 from different metazoan species (human: *Homo sapiens*; danre: *Dano rerio*; galga: *Gallus gallus*; xenle: *Xenopus leavis*; drome: *Drosophila melanogaster*; apime: *Apis mellifica*; culpi: *Culex pipens*; Bruma: *Brugia malayi*; caeel: *Caenorhabditis elegans*). Arrowheads indicate the predicted RNA binding site and asterisks the newly identified dimerization interface. Residue numbering corresponds to the human RBPMS2 sequence. The figure was made using ESPRIPT (http/espript.ibcp.fr). Overall structure of wild-type human RBMPS2-Nter homodimers (**D**) and detailed view of the dimerization interface (**E**) seen in a ribbon diagram. The secondary structure and loops involved in the dimerization are shown as red-to-blue (subunit A) and orange (subunit B) ribbons. Red indicates highly conserved amino acid where blue labels less conserved residues. The side chains that stabilize the dimeric interface are shown as sticks using the CPK color convention. Residues are numbered according to the human RBMPS2 sequence. The figures were prepared using Pymol (http://pymol.sourceforge.net). (**F**) Experimental small-angle X-ray scattering curve (logarithm of intensity in arbitrary units as a function of the momentum transfer range *s* in Å^−1^) for RBPMS2-Nter measured at 1.9 mg/ml (green crosses), with its fitting theoretical curve (red continuous line) back-calculated from the RBPMS2-Nter NMR structure (Supplementary Figure S2). Blue dots represent the relative error bound. The χ^2^ value of the fit is 1.115.

To identify the structural features that are responsible for RBPMS2 function, we analyzed RBPMS2 sequence and its predicted structure. Basic Local Alignment Search Tool (BLAST) searches using human RBPMS2 as a query (NP_919248.1) identified its vertebrate orthologs with high sequence conservation (E-values ranging from 10^−153^ to 10^−114^) and also its closely related paralog RBPMS1 (E-values from 10^−94^ to 10^−74^) (Figure 1A). This analysis also highlighted the strong similarities of human RBPMS2 and insect CPO (E-values from 10^−40^ to 10^−38^) and of both RBPMS2 and CPO sequences with *C. elegans* MEC-8 (∼10^−30^). These three proteins were the first RRM-containing proteins detected by BLAST, suggesting a common evolutionary history and some functional homology. Indeed, no other human RNA-binding protein showed such a strong similarity with RBPMS2 (see the snRNP proteins with E-values ∼10^−3^). Similar results were obtained when we used only the sequence of the RRM domain of RBPMS2, suggesting that RBPMS2, CPO and MEC-8 form an original subfamily of RRM-containing proteins (Figure 1B). This family also includes their paralogs (RBPMS1) that share highly similar RRM sequences in vertebrates. Their common N-terminal RRM domains (90-residue long) share 60–69% of sequence identity. The rest of the sequence showed significant amino acid composition bias toward small and/or polar residues (G, A, S, T, P, Q) and rapid divergence among the closely homologous sequences while no significant similarities could be found with known protein structures. In agreement, disordered segments are predicted by various bioinformatic tools implemented in the meta-server MetaDisorder (32) around the RRM domain (data not shown).

Analysis of the structural organization of human RBPMS2 by molecular modeling using the server @TOME-2 (33) confirmed that its N-terminus (residues 27–117) can fold into a stable and functional RRM domain that harbors the two conserved motifs for RNA binding (named RNP-1 and RNP-2) (Figure 1C). Further sequence-structure analysis highlighted two other patches of high sequence conservation on the RRM surface. One patch corresponds to the last β-strand and the following loop (residues 102–111). By similarity with other RRMs, this segment is predicted to be involved in RNA binding in complement to the motifs RNP-1 and RNP-2. Indeed, three basic residues are conserved in this motif (R102, K107 and K111). On the contrary, the last conserved motif seems specific to the sub-family of RBPMS2-CPO-MEC-8 proteins as shown using PHI-BLAST (34). This new motif (D-x-K-x-R-E-L-Y-L-L-F: residues 39–51 in human RBPMS2) covers the helix α1 and the preceding loop. This sequence motif is not predicted to participate in the RNA-binding platform, although this cannot be ruled out as recently shown in the case of the noncanonical RRM domain of SRSF1 (35). Nevertheless, this signature suggests that this particular RRM domain might be involved in recognizing another partner or in multimerization. Indeed, some RRMs are involved in protein–protein interactions including self-association (15). The latter often relies on a ‘side-by-side’ interaction to form an extended β-sheet. Alternatively, helix–helix interactions have been observed in a few examples (14) and may occur in several others (36). In some cases, RRMs are no longer involved in RNA-binding module but only in protein–protein interactions (37). Note that worm MEC-8 and some insect CPO possess an additional RRM domain at their C-terminus, but this additional RRM does not harbor the conserved motif detected in the N-terminal RRM while it does contain the two important motifs RNP-1 and RNP-2 for RNA binding. In order to clarify this point, we engaged in a thorough structure–function relationship of RBPMS2.

### A new dimer interface within the RBPMS2 RRM motif

To structurally characterize RBPMS2 RRM domain, we over-expressed and purified the truncated RBPMS2-Nter recombinant protein (residues 27–117, thus including the RRM domain) and examined by size-exclusion chromatography with multi-angle laser light scattering (SEC-MALS). In these experiments, samples are fractionated on a gel-filtration column and the absorbance at 280 nm and the refractive index with the multi-angle laser light scattering of the eluates are together monitored. These enable the weight-average molecular weight (*M_w_*) of species in the eluates to be calculated continuously. As shown, in 20-mM Tris pH 8, 300-mM NaCl buffer at loading concentration of 2 mg/ml, RBPMS2-Nter elutes as one single peak from the gel-filtration column and has a *M_w_* of 21.5 kDa (Supplementary Figure S1). Using NMR spectroscopy approach and ^15^N/^13^C-labeled recombinant RBPMS2-Nter, we solved the high-resolution NMR structure of the folded part of RBPMS2 (Table 1 and Supplementary Figure S2) and confirmed the presence of an RRM domain harboring a flat anti-parallel four-stranded β-sheet (β1–β4) and two α-helices (α1 and α2) that are packed on one side of the β-sheet (Figure 1D). The other face of the β-sheet includes the conserved aromatic residues that are expected to interact with RNA nucleotides. We then confirmed RBPMS2 homodimerization via the RRM domain by ^13^C-filtered NMR experiments (Supplementary Figure S2). The surface area buried within the dimer is ∼800 Å^2^ calculated with PISA (http://www.ebi.ac.uk/msd-srv/prot_int/). Similarly, this dimeric structure was confirmed using SAXS measurements at 1.9 mg/ml (χ^2^_crysol = 1.115; Figure 1F) and at 4.2 mg/ml (data not shown) without noticeable concentration effects. In both cases, only one species appears that perfectly matches the theoretical dimeric NMR structure, indicating the absence of any equilibrium.

Analysis of the dimeric structure obtained with NMR indicated that the main interface involved residues R45, E46, L49 and L50 lying on the surface of helix α1 (Figure 1E and C). In addition, the dimer interface involved residues from two loops (residues D41-K43 and I91-P95, respectively) that connect strand β1 to helix α1 and helix α2 to the last β-strand (Figure 1E and F). The dimer was mainly stabilized by hydrophobic interactions between L49/L49′, L49/L50′, L49/F93′, Y48/I91′, Y48/F93′ and Y48/P95′ and electrostatic interactions between D41/K43′ and R45/E46′ (where the symbol ′ means a residue from the second and facing subunit) (Figure 1E). Sequence-structure analysis then identified a new dimerization motif D-x-K-x-R-E-L-Y-L-L-F (residues 39–51 in human RBPMS2) that appears to be conserved (red residues in helix α1) and specific to RBPSM2 orthologs, including CPO and the N-terminal RRM of MEC-8 (Figures 1C–E), while it could not be detected in other proteins harboring RRM domain. We decided to characterize further the functional importance of this new signature of RBPMS2 as well as the importance of this self-association in living cells.

### RBPMS2 forms homodimers *in vitro* and *in vivo*

**Figure 2.**
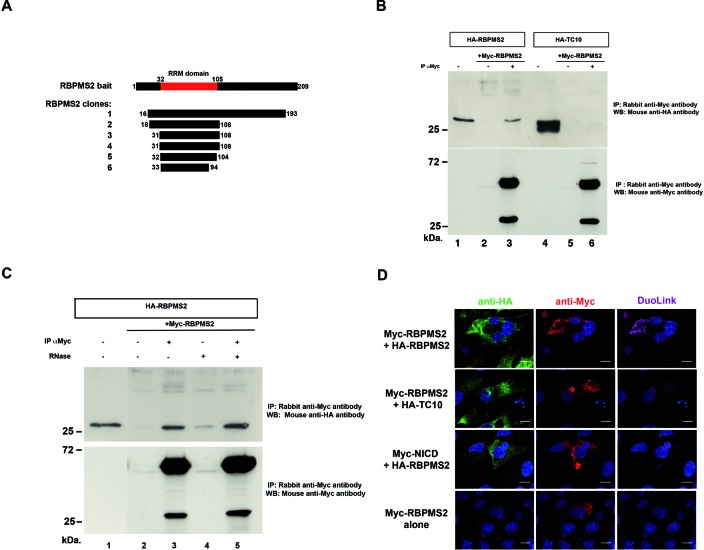
RBPMS2 dimers are formed *in vitro* and *in vivo* independently of RBPMS2 interaction with RNA. (**A**) Schematic representation of the different RBPMS2 clones isolated by Y2H screening with human RBPMS2 as bait. The RRM domain of RBPMS2 is between amino acids 32 and 105. (**B**) Immunoprecipitation with rabbit anti-Myc antibodies (lanes 3 and 6) or without (lanes 2 and 5) of protein lysates from DF-1 cells that express human HA-RBPMS2 or HA-TC10 and Myc-RBPMS2 or not. Lanes 1 and 4: 10% of total protein extracts from cells that express only HA-RBPMS2 or HA-TC10. Co-immunoprecipitation of HA-RBPMS2 was monitored by immunoblotting with mouse anti-HA antibodies (upper panel). The efficiency of immunoprecipitation was monitored by immunoblotting with rabbit anti-Myc antibodies (lower panel). (**C**) Co-immunoprecipitation of HA-RBPMS2 and Myc-RBPMS2 dimers in the absence of RNA. Protein lysates from DF-1 cells that express HA-RBPMS2 alone or with Myc-RBPMS2 (lanes 2–5) were incubated with 50-μg/ml RNase A at room temperature for 30 min (lanes 4 and 5) or left untreated (lanes 1–3) and then immunoprecipitated with rabbit anti-Myc antibodies (lanes 3 and 5) or without (lanes 2 and 4). Lane 1: 10% of total cell extracts from cells that express only HA-RBPMS2. Co-immunoprecipitation was monitored by immunoblotting with mouse anti-HA antibodies (upper panel). The immunoprecipitation efficiency was monitored by immunoblotting with rabbit anti-Myc antibodies (lower panel). (**D**) Analysis of the interaction of Myc-RBPMS2 with HA-RBPMS2 by proximity ligation assays (PLAs) in DF-1 cells that express Myc-RBPMS2 with HA-RBPMS2 or HA-TC10, or Myc-NICD and HA-RBPMS2, or Myc-RBPMS2 alone. HA-tagged proteins were detected with anti-mouse HA antibodies (in green) and Myc-tagged proteins with anti-rabbit Myc antibodies (in red). Interactions between proteins were detected with Duolink PLA labeled in magenta. Images were collected by confocal microscopy. Bars, 10 μm.

First, we carried out a Y2H screen using full-length human *RBPMS2* as bait against a human placenta library. We isolated eight different clones that corresponded to six RBPMS2 sequences. All included a common RBPMS2 region within residues 32 and 105, indicating that the self-interaction region was located in the RRM domain (Figure 2A). We next examined whether RBPMS2 self-associated *in vitro* by co-immunoprecipitation assays (co-IP) using lysates of DF-1 cells that express HA-tagged human RBPMS2 or the small GTPase TC10 protein fused to the HA-tag (negative control) with or without Myc-tagged RBPMS2. HA-RBPMS2, but not HA-TC10, co-precipitated with Myc-RBPMS2 (Figure 2B). In addition, RNase treatment did not affect co-IP of HA-RBPMS2 and Myc-RBPMS2, indicating that this interaction is specific and direct and does not require any RNA molecule (Figure 2C). Then, by using the *in situ* PLA (DuoLink technology) we showed that in DF-1 cells that co-express Myc- and HA-RBPMS2, the interaction between HA-RBPMS2 and Myc-RBPMS2 occurred in the cytoplasm. As negative controls, we also tested and confirmed the absence of interaction between RBPMS2 with the unrelated Myc-NICD or HA-TC10 proteins (Figure 2D). These findings demonstrate that RBPMS2 is a subunit, which self-associates in the cytoplasm through the RRM domain and independently of any RNA.

### A conserved leucine in RBPMS2 RRM is essential for its homodimerization

In order to investigate the functional involvement of RBPMS2 homodimerization and based on the results of the NMR analysis, we decided to alter the homodimerization of RBPMS2 through essential amino-acid substitution in this interface without impacting the overall stability and structure of the RRM domain. If all positions of the motif are deeply involved in this interface, only a few positions can be substituted by residues without simultaneously impacted the overall stability. For this reason, we decided to experimentally focus on the hydrophobic leucine (L) residue (L49 in human and L40 in chick RBPMS2) buried at the center of the subunit–subunit interface essential for RBPMS2 dimerization. We mutated this leucine into an isosteric, negatively charged glutamic acid (E) or polar and neutral glutamine (Q). Indeed, L49E (but not L49Q) substitution is predicted to prevent RBPMS2 homodimerization by charge repulsion without altering the local and global structure of the subunit as L, Q and E residues would favor the helical conformation observed in the NMR structure.

**Figure 3.**
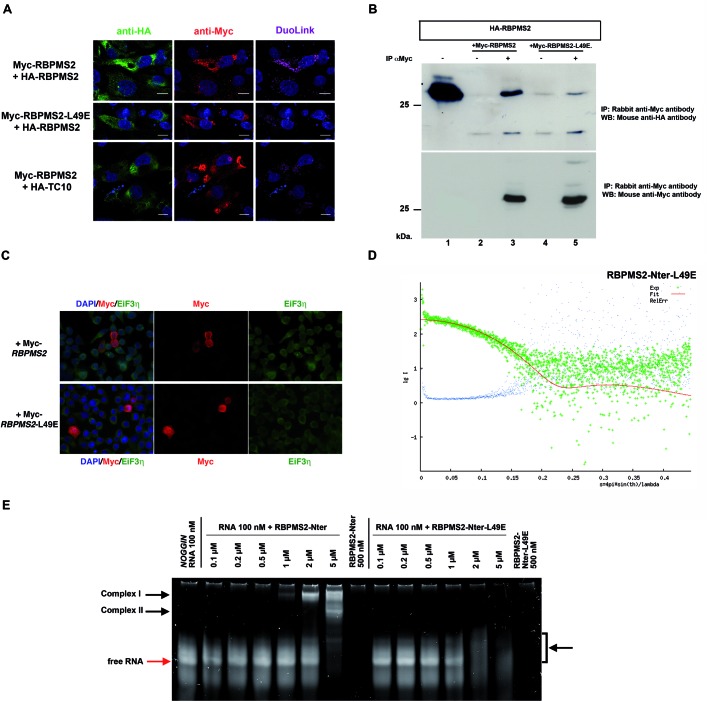
The mutation of Leucine 49 into Glutamic acid (L49E) in the RRM of human RBPMS2 inhibits homodimerization, but does not alter binding to *NOGGIN* mRNA. (**A**) Analysis of the interaction of Myc-RBPMS2 with HA-RBPMS2 by Duolink PLA in DF-1 cells that co-express Myc-RBPMS2 or Myc-RBPMS2-L49E and HA-RBPMS2, or Myc-RBPMS2 and HA-TC10. Ha-tagged proteins were detected with anti-mouse HA antibodies (in green) and Myc-tagged proteins with anti-rabbit Myc antibodies (in red). Protein interactions were detected with Duolink PLA labeled in magenta. Images were collected by confocal microscopy. Bars, 10 μm. (**B**) Immunoprecipitation of RBPMS2 homodimers. Protein lysates from DF-1 cells that express HA-RBPMS2 and Myc*-*RBPMS2 (lanes 2 and 3) or Myc-RBPMS2-L49E (lanes 4 and 5) were immunoprecipitated with rabbit anti-Myc antibodies (lanes 3 and 5) or without (lanes 2 and 4). Lane 1: 10% of total cellular extracts from cells that express HA-RBPMS2 alone. Co-immunoprecipitation was monitored by immunoblotting with mouse anti-HA antibodies (upper panel). Immunoprecipitation efficiency was monitored by immunoblotting with rabbit anti-Myc antibodies (lower panel). (**C**) Subcellular localization of human RBPMS2 and RBPMS2-L49E. HEK293 cells that express Myc-RBPMS2 or Myc-RBPMS2-L49E were detected with anti-EiF3n (eukaryotic translation initiation factor 3n is present in stress granule) and rabbit anti-Myc antibodies. Myc-RBPMS2 and Myc-RBPMS2-L49E show similar cytoplasmic localization. (**D**) Experimental small-angle X-ray scattering curve (logarithm of intensity in arbitrary units as a function of the momentum transfer range *s* in Å^−1^) for RBPMS2-Nter-L49E measured at 1.1 mg/ml (green crosses), with its fitting theoretical curve (red continuous line) back-calculated from the RBPMS2-Nter-L49E NMR structure (Supplementary Figure S2). Blue dots represent the relative error bound. The χ^2^ value of the fit is 1.096. (**E**) EMSA binding assays using a fixed high concentration of 161-nt *NOGGIN* RNA (100 nM) were performed with increasing concentrations of RBPMS2-Nter and RBPMS2-Nter-L49E ranging from 0.1 to 5 μM on a same gel and detected with SYBR^®^ Green EMSA nucleic acid gel stain. Note that RBPMS2-Nter forms defined RNA/protein complex as soon as 1 μM and two complexes at 5 μM (complexes I and II), whereas RBPMS2-Nter-L49E forms very diffuse bands (bracket and arrow). Free 161-nt RNA is indicated with a red arrow.

We first screened these RBPMS2 mutant forms using PLA approach and we showed that in DF-1 cells that co-express Myc-RBPMS2-L49E and HA-RBPMS2, the interaction between RBPMS2 and RBPMS2-L49E is strongly decreased (Figure 3A). In contrast, the interaction between RBPMS2 and RBPMS2-L49Q is similar to that observed with wild-type RBPMS2 (Supplemental Figure S3A). We also analyzed these interaction using co-IP assays with anti-Myc antibodies in lysates from DF-1 cells that express HA-RBPMS2 and Myc-RBPMS2-L49E and found that L49E substitution decreased by 87% the interaction of RBPMS2 strongly altering the capacity of RBPMS2 to homodimerize (Figure 3B). In order to identify a potential instability or cellular mislocalization of RBPMS2-L49E protein, we analyzed its cellular localization and found that both RBPMS2 and RBPMS2-L49E were detected in stress granules where RNA granules are also localized (Figure 3C).

Then, we confirmed by NMR that the RRM fold of RBPMS2-Nter-L49E was not affected by this specific mutation (Supplementary Figure S2). By SEC-MALS, we found that RBPMS2-Nter-L49E at loading concentration of 4 mg/ml elutes in two peaks from the gel-filtration column. The majority species (92.4%) has a *M_w_* of 10.6 kDa, as would be expected for the monomer, where the minority species has a *M_w_* of 19.4 kDa (Supplementary Figure S1). SAXS data at low concentration (1.1 mg/ml) showed that only one species appears, which perfectly matches the theoretical monomeric NMR structure (χ^2^_crysol = 1.096; Figure 3D). However, a slight monomer-dimer equilibrium was detected for the RBPMS2-Nter-L49E at higher concentration (2.2 mg/ml; data not shown). Indeed, inspection of the low-angle region of the curves indicates the presence of slight attractive interparticle interactions that were minimized when merging the curves, consolidating the results observed in SEC-MALS experiment (Supplementary Figure S1).

In agreement with our previous finding that chick RBPMS2 interacts with *NOGGIN* mRNA (9), we observed by fluorescence anisotropy-based binding assays that the recombinant human RBPMS2-Nter protein bound to human *NOGGIN* mRNA *in vitro* (Supplementary Figure S4). Furthermore, this assay led us to identify the region located between nucleotides 570–730 of *NOGGIN* mRNA (referred to as 161-nt *NOGGIN* RNA) as the specific binding region recognized by RBPMS2-Nter. We show that the 161-nt *NOGGIN* RNA is also recognized by the monomeric mutant RBPMS2-Nter-L49E (Supplementary Figure S4). In order to provide a qualitative indicator of the binding capacity of both RBPMS2-Nter and RBPMS2-Nter-L49E proteins to target 161-nt *NOGGIN* RNA, we performed protein/RNA binding interaction using EMSAs in native 10% polyacrylamide gels, run at 4°C. Increasing concentrations of each proteins were added to constant 100-nM 161-nt *NOGGIN* RNA and both RNA and protein migrations were evaluated on the same gel (Figure 3E). We found that RBPMS2-Nter starts to bind 161-nt *NOGGIN* RNA at 1 μM and complexes almost all RNA at 5 μM in a well-defined manner, with a theoretical *K_d_* at 2.25 ± 0.09 μM (Figure 3E and Supplementary Figure S5). Moreover a second RNA–protein complex appears at 5 μM and co-migrates with RBPMS2-Nter protein further than the first complex (Supplementary Figure S5), suggesting a more compact rearrangement of either RNA or protein at this concentration. In contrast, in the presence of RBPMS2-Nter-L49E we find that the free 161-nt *NOGGIN* RNA starts to be bound at 1 μM and thereafter decreases, forming a broader and less intense smear at 2 and 5 μM instead of the original free RNA. However, no any shifted complex is visible in the corresponding migration zone where the RNA/RBPMS2-Nter complexes were observed (Figure 3E and Supplementary Figure S5), so that we were not able to establish any theoretical *K_d_* for RBPMS2-Nter-L49E. Nevertheless, such binding mode suggests that the complexes are heterogeneous, with RBPMS2-Nter-L49E binding at different position on the target RNA as observed for PTB1 protein (38). EMSA experiments illustrate apparent variations in binding modes and apparent affinities of RBPMS2-Nter and RBPMS2-Nter-L49E proteins. These findings indicate that substitution of leucine into glutamic acid at position 49 within the RRM domain prevents RBPMS2 self-association and does not alter RBPMS2 protein folding but changes its behavior to bind target RNA.

### RBPMS2 homodimerization is crucial for its biological function

**Figure 4.**
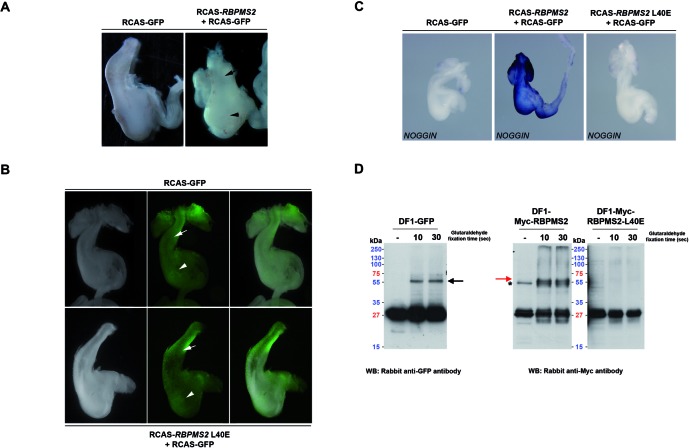
RBPMS2 homodimerization is required for RBPMS2 function. (**A**) Stomachs from E9 chicken embryos after retroviral misexpression of RCAS-GFP alone (negative control) or of RCAS-*RBPMS2* and RCAS-GFP. The presence of retroviruses was confirmed by direct observation of GFP expression. Sustained expression of chicken RBPMS2 leads to specific stomach malformations: hypertrophied proventriculus (arrows) and denser and malformed gizzard (arrowheads). (**B**) Stomachs from E9 chicken embryos after retroviral misexpression of RCAS-GFP (control) or of the RCAS-*RBPMS2*-L40E mutant that cannot homodimerize with RCAS-GFP. White arrows and arrowheads indicate, respectively, the gizzard and the proventriculus. (**C**) Whole-mount *in situ* hybridization analysis of *NOGGIN* expression in stomachs from E9 chicken embryos after retroviral misexpression of RCAS-GFP (control), RCAS-*RBPMS2* and RCAS-GFP, or RCAS-*RBPMS2*-L40E and RCAS-GFP. Infection was confirmed by direct observation of GFP expression. (**D**) Glutaraldehyde crosslink assay of protein extract from DF1 cells infected either with RCAS-GFP (positive control, left panel), RCAS-Myc-*RBPMS2* or RCAS-Myc-*RBPMS2*-L40E (right panels), followed by SDS-PAGE separation and revealed by rabbit anti-GFP or anti-Myc antibodies. Anti-GFP antibody revealed GFP monomers and dimers (near 55 kDa, black arrow), as expected. Anti-Myc antibody revealed Myc-RBPMS2 monomers at 27 kDa on both Myc-RBPMS2 and Myc-RBPMS2-L40E expressing cell protein extract, but only Myc-RBPMS2 expressing cell protein extract presents Myc-RBPMS2 dimerization at 55 kDa (red arrow). Note the below 50-kDa bands (asterisk) on Myc-RBPMS2 expressing cell protein extract correspond to the induction of endogenous avian MYC protein.

To further investigate whether RBPMS2 homodimerization has a role in its biological function, we expressed chick RBPMS2 or the chick mutant of human RBPMS2-L49E (RBPMS2-L40E) in the gastrointestinal mesenchyme of stage-10 chicken embryos using an avian replication-competent retroviral misexpression system (10,18). In accordance with our previous results (9), sustained *RBPMS2* expression resulted in a dramatic alteration of the stomach morphology: the proventriculus, which is the glandular part of the chick stomach, was hypertrophied and the gizzard was denser and malformed in comparison to controls that overexpressed GFP alone (Figure 4A). In contrast, sustained expression of RBPMS2-L40E did not induce any morphological change (*n* = 26, stomachs; Figure 4B). Moreover, while misexpression of wild-type RBPMS2 in the gastrointestinal mesenchyme upregulated *NOGGIN* mRNA expression in comparison to controls (Figure 4C, left and middle panels), misexpression of *RBPMS2*-L40E did not (*n* = 17 infected stomachs; Figure 4C, right panel). Lastly, we verified the homodimerization status of Myc-RBPMS2, Myc-RBPMS2-L40E and GFP proteins in DF1 cell line by glutaraldehyde crosslinking. Interestingly, whereas fixed protein extract from GFP (as positive control) or Myc-RBPMS2 expressing cells displays well-defined homodimeric forms, no clear dimerization was observed in fixed protein extract from Myc-RBPMS2-L40 expressing cells (Figure 4D). Altogether these results demonstrate that RBPMS2 homodimerization is essential for RBPMS2-mediated upregulation of *NOGGIN*.

**Figure 5.**
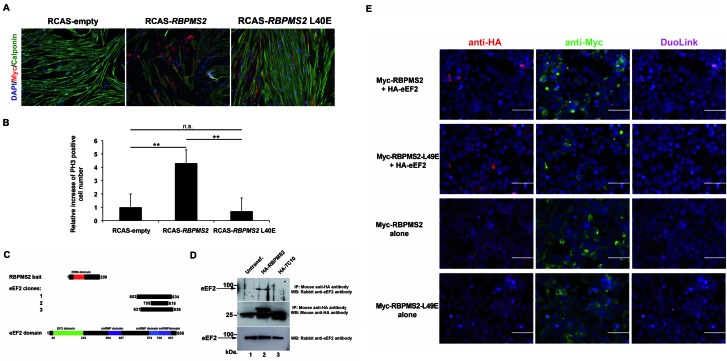
RBPMS2 homodimerization is necessary to dedifferentiate digestive SMCs. (**A**) Immunofluorescence analysis of primary SMC cultures infected with RCAS-empty, RCAS-Myc-*RBPMS2* and RCAS-Myc-*RBPMS2*-L40E retroviruses for 3 days. Nuclei were visualized with Hoechst. Anti-Calponin antibodies were used as a marker of SMC differentiation and anti-Myc antibodies to identify cells infected by retroviruses that express chicken *RBPMS2* or *RBPMS2*-L40E. (**B**) Quantification of mitotic cells using anti-phosphorylated Histone 3-Ser10 (PH3) antibodies in primary SMC cultures infected with retroviruses that express *RBPMS2* or *RBPMS2*-L40E retroviruses or controls (RCAS-empty) for 7 days. Values are the mean ± standard error of the mean of two independent experiments (RCAS-*RBPMS2* or RCAS-*RBPMS2* L40E versus RCAS-empty). **for *P* < 0.01; n.s. for no statistically significant. (**C**) Schematic representation of the *eEF2* clones isolated by Y2H screening with human RBPMS2 as bait. Human eEF2 harbors one elongation factor domain (EF2) and three U5 small nuclear ribonucleoprotein domains (snRNP). (**D**) Immunoprecipitation with mouse anti-HA antibodies of protein lysates from HEK293 cells that express human HA-RBPMS2, HA-TC10 or not. Co-immunoprecipitation of endogenous eEF2 was monitored by immunoblotting with rabbit anti-eEF2 antibodies (upper panel). The efficiency of immunoprecipitation of HA-RBPMS2 and HA-TC10 was monitored by immunoblotting with mouse anti-HA antibodies (middle panel). Lower panel: 10% of total protein extracts from cells of each condition monitored by immunoblotting with rabbit anti-eEF2 antibodies. (**E**) Analysis of the interaction of Myc-RBPMS2 with HA-eEF2 by Duolink PLA in HEK293 cells that co-express Myc-RBPMS2 or Myc-RBPMS2-L49E and HA-eEF2. HA-eEF2 was detected with anti-mouse HA antibodies (in red) and Myc-tagged proteins with anti-rabbit Myc antibodies (in green). Protein interactions were detected with Duolink PLA labeled in magenta. Bars, 50 μm.

We then asked whether RBPMS2 homodimerization was required also for regulating SMC plasticity. To this aim, we established primary cultures of digestive differentiated SMCs in serum-free medium supplemented with insulin and BSA as previously published (9). After 3 days, in control SMC cultures, Calponin, an SMC contractile marker, was homogeneously expressed in highly organized filament bundles and cells were spindle-shaped (Figure 5A; Supplementary Figure S6). Conversely, in cells that express wild-type RBPMS2, Calponin expression was lost. Interestingly, SMCs expressing RBPMS2-L40E present no decrease of Calponin expression compared to control (Figure 5A). Moreover, after 6 days of culture, the number of cells that expressed phosphorylated histone 3-Ser10 (PH3), a standard marker of G2/M transition, was 4.5-fold higher in cultures that overexpress wild-type RBPMS2 than in control cells and SMC cells that express RBPMS2-L40E (Figure 5B), as also observed in heterologous DF1 cell line (Figure 4D). In addition, we also compared the impact of human RBPMS2, RBPMS2-L49E and L49Q in primary SMC culture and found that RBPMS2-L49Q like RBPMS2 induced an alteration of Calponin expression, whereas RBPMS2-L49E not (Supplementary Figure S3B). These results demonstrate that RBPMS2 homodimerization is essential to induce SMC dedifferentiation and proliferation.

In order to better characterize the difference of action between dimeric wild-type RBPMS2 and monomeric mutant RBPMS2-L49E proteins, we came back to our Y2H screen using human *RBPMS2* as bait and identified three different clones that correspond to the eukaryote elongation factor-2 (eEF2) (Figure 5C). eEF2 is essential for the translational elongation of specific neuronal mRNAs (39), but eEF2 is also involved in the delay protein synthesis during muscular exercise through its specific inhibitory phosphorylation (40). We next examined whether RBPMS2 and eEF2 interact *in vitro* by co-IPs using lysates of HEK293 cells that express HA-tagged human RBPMS2 or the small GTPase TC10 protein fused to the HA-tag (negative control). HA-RBPMS2, but not HA-TC10, co-precipitated with endogenous human eEF2 (Figure 5D). Then, by using the *in situ* PLA (DuoLink technology) we showed that in HEK293 cells that co-express Myc-RBPMS2 and HA-eEF2, the interaction between Myc-RBPMS2 and HA-eEF2 occurred in the cytoplasm (Figure 5E). In addition, we showed that in HEK293 cells that co-express Myc-RBPMS2-L49E and HA-eEF2, the interaction between RBPMS2-L49E and eEF2 is strongly decreased (Figure 5E). These findings indicate that homodimerization of RBPMS2 is required to interact with translational elongation factor eEF2.

## DISCUSSION

Previous studies demonstrated that post-transcriptional events control the development and the plasticity of different types of smooth muscles through miR or RNA-binding protein expression and function (2,3,7,8,9,11). Among these important players is the RNA-binding protein RBPMS2, which is involved in digestive SMC development and plasticity through the control of BMP signaling activity (9,11). Indeed, RBPMS2 is an early marker of digestive SMC precursors and its ectopic expression leads to the dedifferentiation of digestive mature SMCs showing that RBPMS2 controls both development and plasticity of digestive SMCs (9,10). In addition, aberrant elevated expressions of RBPMS2 were specifically observed in digestive myopathy syndrome (CIPO) and gastrointestinal mesenchymal neoplasm (GIST), demonstrating that RBPMS2 expression and function must be tightly regulated to avoid SMC dedifferentiation (9,11).

Our previous work demonstrates that the avian RBPMS2 protein forms ribonucleoprotein complex with *NOGGIN* mRNA (9). In this study, we showed that human RBPMS2 is able to directly form an RNA/protein complex with human *NOGGIN* RNA demonstrating conservation of RBPMS2-target RNA.

Here, we show using multiple approaches (NMR, SAXS, SEC-MALS, co-immunoprecipitation, *in cellulo* glutaraldehyde crosslinking, Y2H and PLA DuoLink) that vertebrate RBPMS2 protein forms stable dimers *in vitro* and *in vivo*. This dimer is formed via the RRM domain through a specific sequence motif or signature (^39^D-x-K-x-R-E-L-Y-L-L-F^51^ in human RBPMS2). This motif is conserved only in related proteins, such as RBPMS1, insect CPO and worm MEC-8. This conservation is in agreement with the proposed self-interaction of MEC-8 previously detected in a large-scale study (41). Nevertheless, this study did not highlight the interface responsible of this self-interaction. It would be of great interest to investigate this interaction in worm MEC-8 and insect ortholog CPO to confirm the importance of such novel interaction sequence through evolution, thus establishing this motif as a signature of a novel subfamily of RNA-binding proteins. But the sequence conservation and the common involvement in nerve and muscle development of MEC-8, CPO, RBPMS and RBPMS2 makes them likely orthologous sequences belonging to an original sub-family of the RRM-containing proteins. In agreement, with this proposed orthology, it was recently shown that these RRMs recognized similar RNA motifs containing a CAC triplet (31,42).

Interestingly, analysis of the human *NOGGIN* mRNA and its avian homolog highlights the presence of CAC motif in tandem (from positions 86 to 97 of human *NOGGIN* cDNA). In this case, the two recognized motifs are separated by 6 nt, which is consistent with one dimeric RBPMS2 binding the *NOGGIN* RNA. While the monomeric RBPMS2 can still bind RNA with similar affinity, EMSA experiments revealed that it induces a smeary band, suggesting the lack of supramolecular organization in the resulting ribonucleoprotein complex. EMSA experiments were correlated with our *in vivo* experiments, where monomeric RBPMS2 is unable to induce *NOGGIN* expression compared to RBPMS2. Our experiments then clearly demonstrated that RBPMS2 homodimerization is essential for its capacity to induce *NOGGIN* mRNA accumulation *in vivo* and to trigger the SMC dedifferentiation process.

**Figure 6.**
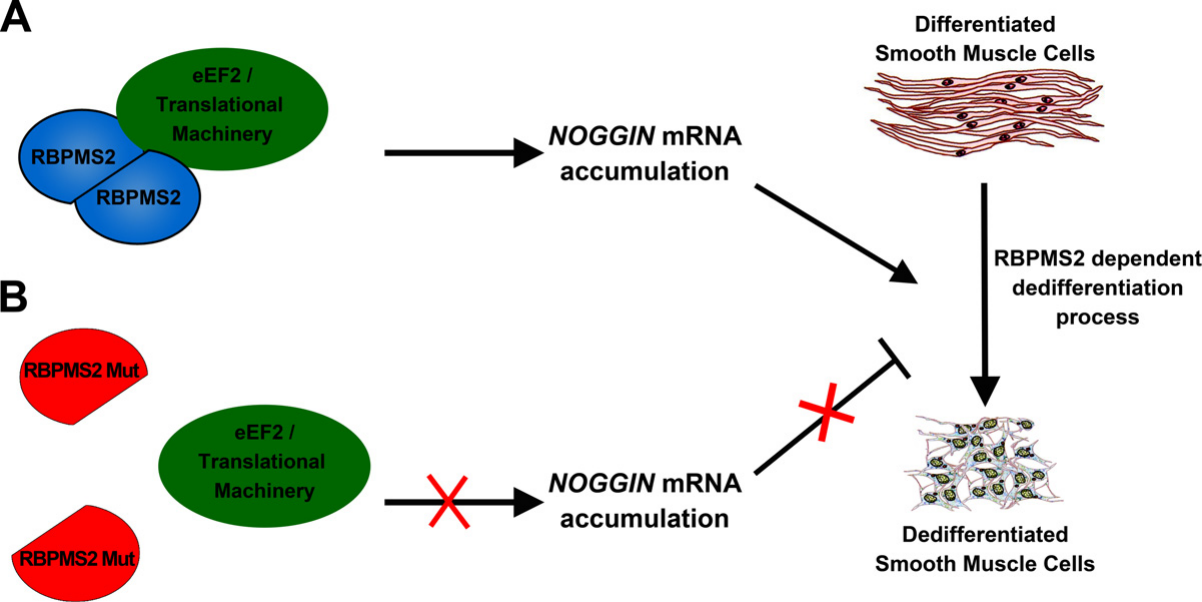
Model of the action of RBPMS2 homodimerization in the SMC dedifferentiation process. Homodimeric RBPMS2 complex interacts with eEF2, an essential member of the eukaryote translational machinery that allows the accumulation of *NOGGIN* mRNA that will be further translated to inhibit BMP pathway activity (9) and to trigger the dedifferentiation of the SMCs (**A**). Disruption of RBPMS2 homodimerization through Leu to Glu substitution in position 49 leads to monomeric RBPMS2 proteins unable to interact with eEF2 and inefficient to induce the dedifferentiation process (**B**).

In addition, we also found that dimeric RBPMS2 interacts with elongation factor eEF2, whereas monomeric RBPMS2 does not. Altogether, our experiments drive us to propose a model in which dimeric RBPMS2 could recruit specific protein partner(s) such as translational factor eEF2 in order to connect RBPMS2 and *NOGGIN* mRNA to the translational machinery complex to promote the SMC dedifferentiation process (Figure 6A). On the opposite way, monomeric RBPMS2 subunit is unable to interact with partners such as eEF2 and to allow the accumulation of *NOGGIN* mRNA (Figure 6B).

In conclusion, using integrative approaches for studying *in vivo* protein–protein interaction and sequence–structure relationships, we have identified a specific homodimerization motif in RBPMS2 RRM-domain and have demonstrated the functional requirement of its homodimerization to drive the RBPMS2-dependent SMC dedifferentiation process. Understanding how RBPMS2 activity is regulated could therefore be useful to develop new therapeutic strategies to target and inhibit RBPMS2 function in digestive-associated pathologies.

## ACCESSION NUMBER

The coordinates of the RRM domain of RBPMS2 have been deposited in the Protein Data Bank (PDB), www.pdb.org (PDB ID code 2M9K).

## SUPPLEMENTARY DATA

Supplementary Data are available at NAR Online.
